# Sponges of Carboxymethyl Chitosan Grafted with Collagen Peptides for Wound Healing

**DOI:** 10.3390/ijms20163890

**Published:** 2019-08-09

**Authors:** Yu Cheng, Zhang Hu, Yuntao Zhao, Zuhao Zou, Sitong Lu, Bijun Zhang, Sidong Li

**Affiliations:** 1 Faculty of Chemistry and Environment Science, Guangdong Ocean University, Zhanjiang 524088, China; 2 College of Food Science and Technology, Guangdong Ocean University, Zhanjiang 524088, China

**Keywords:** carboxymethyl chitosan, collagen peptides, burns, wound healing

## Abstract

Burns are physically debilitating and potentially fatal injuries. Two marine biomaterials, carboxymethyl chitosan (CMC) and collagen peptides (COP), have emerged as promising burn dressings. In this paper, sponges of carboxymethyl chitosan grafted with collagen peptide (CMC–COP) were prepared by covalent coupling and freeze drying. Scanning electron microscopy (SEM) and Fourier transform infrared (FTIR) spectroscopy were then used to characterize the prepared sponges. To evaluate the wound healing activity of the CMC–COP sponges, in vitro tests including cell viability scratch wound healing and scald wound healing experiments were performed in rabbits. Appearance studies revealed the porous nature of sponges and FTIR spectroscopy demonstrated the successful incorporation of COP into CMC. The in vitro scratch assay showed that treatment with CMC–COP sponges (at 100 μg/mL) had significant effects on scratch closure. For burn wounds treated with CMC–COP, regeneration of the epidermis and collagen fiber deposition was observed on day 7, with complete healing of the epidermis and wound on days 14 and 21, respectively. Based on the pathological examination by hematoxylin and eosinstaining, the CMC–COP group demonstrated pronounced wound healing efficiencies. These results confirmed that the CMC–COP treatment enhanced cell migration and promoted skin regeneration, thereby highlighting the potential application of these sponges in burn care.

## 1. Introduction

Burns are one of the most common injuries in daily life. The occurrence of superinfection is a major problem in the management of burns, causing secondary physical and psychological damage to patients. In recent years, extensive studies have developed new biodegradable dressings that promote wound healing. Chitosan and its derivatives have attracted considerable interest due to their ability to accelerate wound healing at the molecular, cellular, and systemic levels [1].

Carboxymethyl chitosan is prepared by the introduction of a carboxymethyl group from chloroacetic acid to the -OH and -NH_2_ groups in chitosan. This results in an increase in the water solubility and pH sensitivity of carboxymethyl chitosan, making it a widely used chitosan derivative in the biomedical field [2]. It is highly soluble in neutral and alkaline solutions and exhibits better sensitivity, biocompatibility, biodegradability, and moisture-retaining capacity than chitosan alone [3,4]. Moreover, its chemotaxis to neutrophils and macrophages helps prevent wound infection in early healing and is beneficial to granulation tissue formation and epidermal cell regeneration in late healing [5,6]. In addition, carboxymethyl chitosan can reportedly inhibit the formation of algogenic substances, such as histamine, serotonin, and bradykinin, as well as prevent scar formation and promote normal fibrocyte growth [7,8].

Collagen peptides have been shown to promote healing in mouse skin in vitro and in vivo [9]. Furthermore, clinical application of collagen peptides from fish has become an emerging research area. At present, various fish collagen products have been marketed for the hemostasis and healing of trauma, burns, chronic ulcers, and other wounds [10]. Collagen exhibits low inflammation, good biocompatibility, and the ability to promote cell attachment and proliferation [11]. Moreover, it has the potential to be widely used in the treatment of skin wounds due to its low antigenicity, small molecular mass, and easy access [12,13].

An ideal topical agent for burn management should form a rapid film, reduce drainage, show abroad antibacterial spectrum and strong antibacterial activity, demonstrate good analgesic effects, accelerate wound healing, cause few side effects, result in less scarring after healing, and be cost effective. As carboxymethyl chitosan and fish collagen peptides are derived from marine organisms, they are available in large quantity and at low cost, and thus are ideal materials for burn dressings [14]. Although carboxymethyl chitosan is highly brittle and insufficiently flexible and collagen peptides degrade rapidly [15], their mixture can make up for their deficiencies and improve performance. In this study, carboxymethyl chitosan sponges grafted with collagen peptides (CMC–COP) were prepared by chemical coupling and freeze-drying technology. The effects of CMC–COP sponges on wound healing in New Zealand rabbits were investigated and compared with a commercial burn ointment (MEBO) to provide a theoretical basis for its future research and development in medicine.

## 2. Results and Discussion

### 2.1. Amino Acid Composition of COP

High-performance liquid chromatography (HPLC) showed that the average molecular weight of 24.5% of collagen peptides was between 1000 and 2000 Da and 24.6% was between 500 and 1000 Da. Peptides with small molecular weights are highly water soluble and easily absorbed by the skin. Table 1 shows the type and content of amino acids hydrolyzed from collagen peptides. A total of 17 amino acids were identified, among which eight were essential amino acids and nine were non-essential amino acids. Glycine (22.1%) accounted for the highest content, followed by proline (11.3%), which together constituted one-third of total amino acids. The content of imino acid (proline and hydroxyproline) was 22.8%. The antioxidant and tissue regeneration, promoting the activities of collagen peptides, are related to amino acid composition [16,17].

### 2.2. Preparation of CMC–COP Sponges

**Figure 1:**
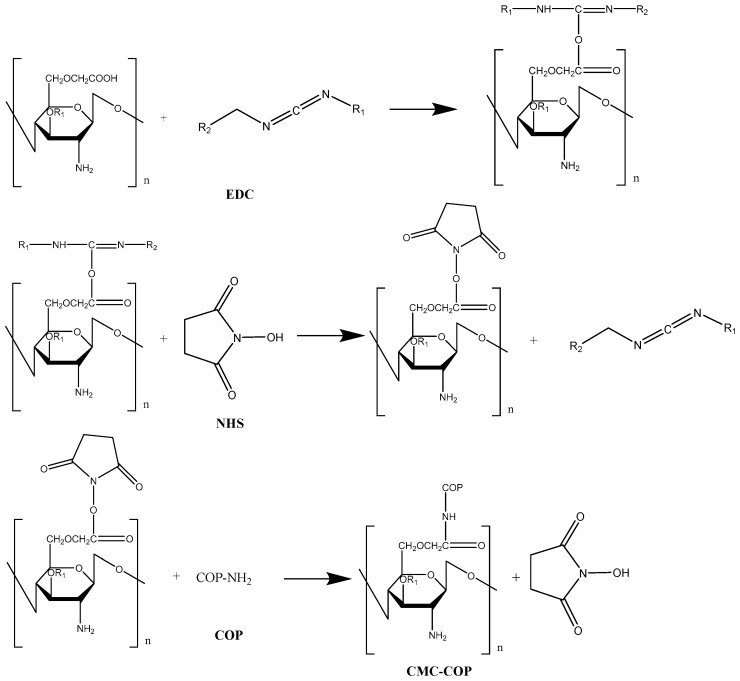
Mechanism of carboxymethyl chitosan grafted with collagen peptide. Carboxymethyl chitosan sponges grafted with collagen peptides (CMC–COP); NHS, N-hydroxysuccinimide.

**Figure 2:**
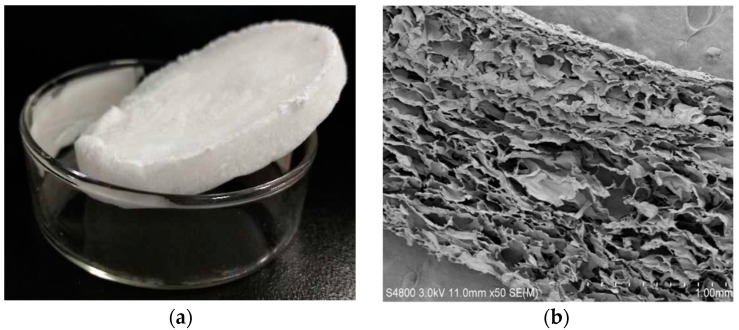
Sponge appearance (digital camera) (**a**) and internal structure (scanning electron microscope) (**b**).

The grafting mechanism of carboxymethyl chitosan with collagen peptide is shown in Figure 1. To prevent hydrolysis of 1-(3-dimethylaminopropyl)-3-ethylcarbodiimide hydrochloride (EDC), and to ensure maximum collagen peptide activity, this reaction was performed in a buffer with a pH of 6.0 [18]. Different mass ratios of EDC to N-hydroxysuccinimide (NHS) have been used in previous studies, most commonly 4:1 and 5:3 [19]. In the present work, a sponge with good toughness was obtained at a mass ratio of 7:3, which was closely related to the molecular weight and amounts of carboxymethyl chitosan and collagen peptide [20]. Samples with good toughness and white color were also spongy (Figure 2a). The internal structure of the sponges was observed by scanning electron microscopy, as shown in Figure 2b. The sponges boasted a porous structure, with pores layered and neatly arranged with uniform size distribution. The softness of collagen offset the poor flexibility of carboxymethyl chitosan. Thus, the composite sponges not only retained the desired biological properties of the two components, but also exhibited enhanced mechanical properties [21]. In addition, the DS of the products can be influenced by the reaction temperature and time [19]. In the present study, carboxymethyl chitosan reacted with collagen at 45 °C for 16 h at mass ratios of 1:4, 1:6, and 1:8, thereby generating sponges with DS values of 12%, 26%, and 58%, respectively (namedCMC–COP12, CMC–COP26, and CMC–COP58, respectively).

### 2.3. FT-IR Analysis

**Figure 3:**
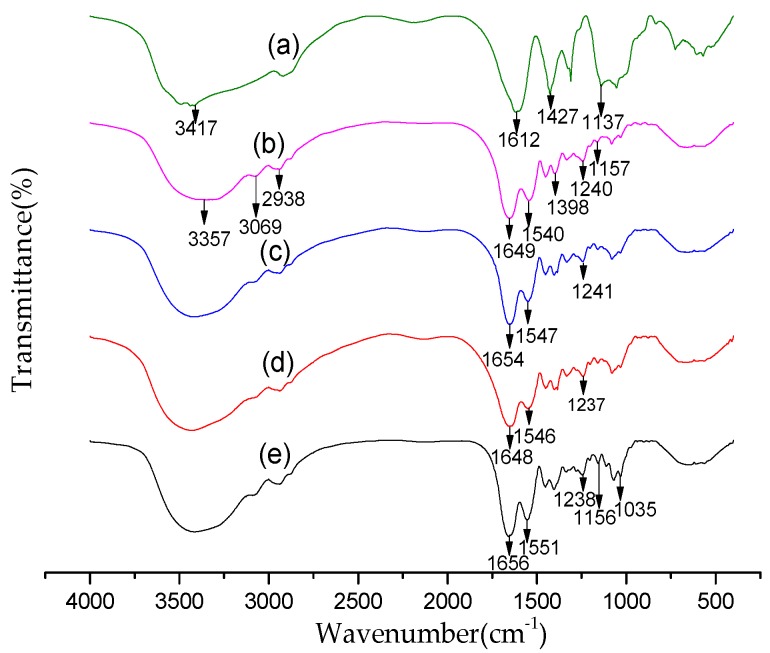
FTIR spectra of (**a**) carboxymethyl chitosan, (**b**) COP, (**c**) CMC–COP12, (**d**) CMC–COP26, and (**e**) CMC–COP58.

Figure 3 shows the FT-IR spectra of carboxymethyl chitosan, collagen peptide, and CMC–COP sponges with different DS. As shown in Figure 3a, the broad band peak at 3417 cm^−1^ was assigned to -OH stretching vibrations of carboxymethyl chitosan, which overlapped with -NH stretching vibrations. Due to this broad peak, there were no obvious differences between the collagen peptide and the three CMC–COP sponge samples. The characteristic peaks at 1612 and 1427 cm^−1^ were assigned to the asymmetric and symmetric stretching vibrations of the carboxyl group of carboxymethyl chitosan, respectively. In Figure 3b, the peaks at 3357 and 3069 cm^−1^ were assigned to the typical amide A and B bands of collagen peptide, respectively. The peak at 2938 cm^−1^ was assigned to the stretching vibration of C–H. The 1649, 1540, and 1240 cm^−1^ peaks were attributed to the amide I, II, and III bands, respectively, which are typical bands of amino groups in collagen [22]. The peaks around 1398 cm^−1^ and 1157 cm^−1^ were assigned to the C–H bending vibration of collagen peptide and asymmetric stretching vibration of -COO–C, respectively. Compared with the collagen peptide, as shown in Figure 3c–e, the three CMC–COP sponge samples had different IR results. The wave patterns of the amide I, II, and III bands of collagen peptide were basically unchanged, but the intensity and positions (located at 1648–1656, 1546–1551, and 1237–1241 cm^−1^, respectively) were slightly altered, thereby confirming the successful grafting of COP to CMCS. Additionally, the peak at 1157 cm^−1^ in Figure 3a was extremely low. The intensity of this peak increased with the increase in substitution degree. The 1137 cm^−1^ peak of carboxymethyl chitosan indicated that -COO–C was involved in the formation of intermolecular forces during crosslinking. The fingerprint region of the CMC–COP spectra was very sensitive to overall structural changes. The strong sharp peak at 1035 cm^−1^ was attributed to the -C–O–C stretching vibration of the backbone [23]. As seen in Figure 3e, the intensity of the peak at 1035 cm^−1^ increased with carboxymethyl chitosan content. Based on the changes in intensity and position of the peaks at different wavenumber, covalent coupling successfully occurred between CMC and COP, with the intensity related to their proportions.

### 2.4. Evaluation of Cell Viability

**Figure 4:**
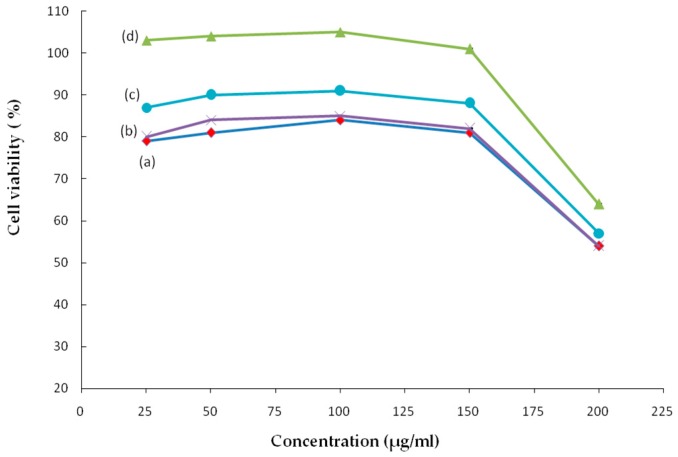
Effect of samples at different concentrations on L929 cell viability: (**a**) CMC, (**b**) CMC–COP12, (**c**) CMC–COP26, and (**d**) CMC–COP58.

Carboxymethyl chitosan itself promoted L929 cell viability due to its sufficient water solubility and excellent biological activity [24,25]. From Figure 4, when the collagen peptide was introduced, high cell viability was observed for the CMC–COP samples (concentrations between 25–200 μg/mL), with CMC–COP58 demonstrating the highest cell viability rate. This indicated that the introduction of collagen peptide enhanced the promotion effects of carboxymethyl chitosan on the viability of L929 cells. Although the specific mechanism of action was unclear, several studies have shown that collagen contains a large number of carboxyl functional groups and amino acid sequences, some of which can be specifically recognized by cells to provide a suitable environment for fibroblast adhesion, proliferation, and differentiation, thus resulting in improved cell viability [26]. Here, cell viability first increased and subsequently decreased with the increase in CMC–COP concentrations. The highest cell viability was observed at 100 μg/mL.

### 2.5. CMC–COP Synergizes the Migration of L929 Cells

**Figure 5:**
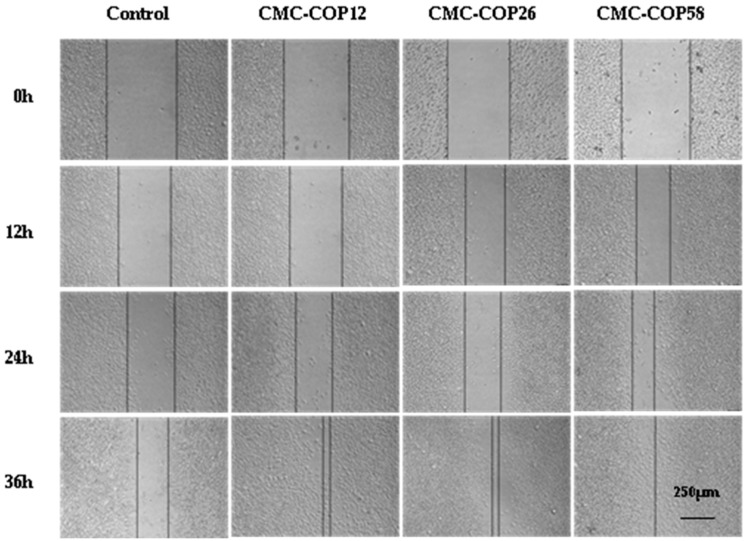
In vitro scratch results of CMC–COP samples.

**Figure 6:**
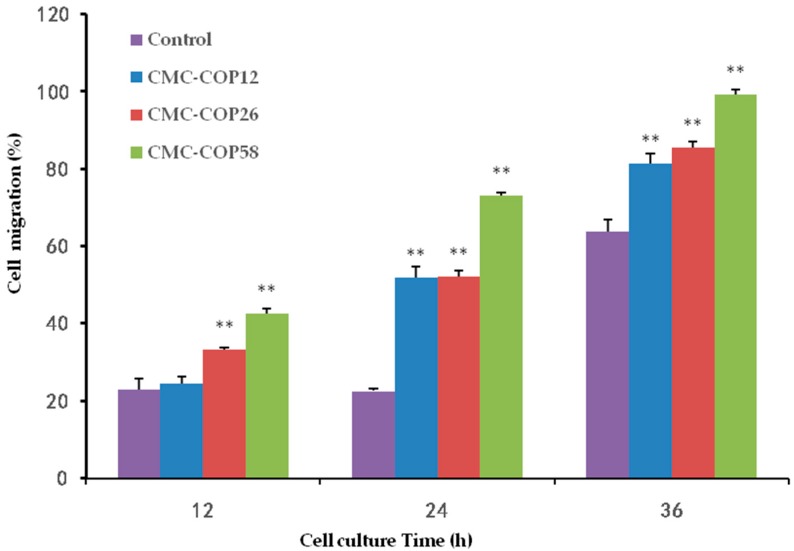
Effect of CMC–COP samples on the L929 cell migration rate (*n* = 6). ** *p* < 0.01.

As shown in Figure 5, at 12 h, oblivious cell migration was observed at the scratch edge, and the widest space was observed in the control group. At 24 h, CMC–COP further narrowed the scratch. At 36 h, the scratch completely disappeared in the CMC–COP58 group, and almost disappeared in the CMC–COP12 and CMC–COP26 groups. As shown in Figure 6, the cell migration rate was significantly higher in the CMC–COP12, CMC–COP26, and CMC–COP58 groups than in the control group (* *p* < 0.01). The highest migration rate occurred in the CMC–COP58 group, consistent with the cell proliferation results described above. Previous studies have shown that carboxymethyl chitosan stimulates the macrophage release of L929 cell growth factor, and living autologous cells subsequently stimulate the migration of L929 and other wound healing cells under the regulation of growth factors and bioactive substances [27]. Moreover, collagen peptides control many cellular functions, including cellular migration. These findings provide preliminary evidence that CMC–COP may promote the migration of L929 cells, which play a major role in the healing of wound surfaces.

### 2.6. Animal Scald Experiment

#### 2.6.1. Macroscopic Analysis of Wounds

**Figure 7:**
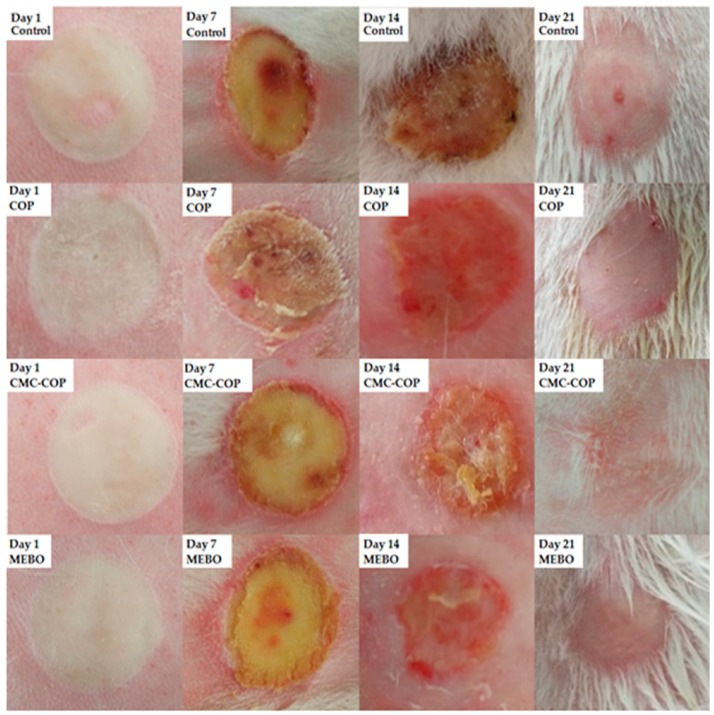
Macroscopic analysis of cutaneous wounds on days 1, 7, 14, and 21 following treatment with COP, CMC–COP, or MEBO (commercial burn cream).

Based on the cell proliferation and cell migration assays, CMC–COP with a DS of 58% was used for the burn-wound healing study. After burn creation on the rabbits, the wound surface became pale and vasospasm occurred (Figure 7). On day 1, edema and whitening around the wound were observed for all rabbits. On day 7, the wound area decreased, and eschars began to form in the healed area in all groups. On day 14, the healed area was light red, eschars became separated in half of the rabbits, and more rapid healing was observed in the CMC–COP and MEBO groups compared to the control and COP groups. On day 21, the eschars became separated in all rabbits. However, red spots remained in the control and COP groups and no hair growth found on the healed wound surfaces. In contrast, a small amount of hair was observed in the CMC–COP and MEBO groups. Macroscopic observation revealed higher moisture retention in the CMC–COP and MEBO groups than in the control and COP groups. Keeping wounds moist can improve healing speed [28]. The moisture retention properties of CMC were found to be quite similar to those of hyaluronic acid. Furthermore, the moisture retention capacity of CMC increased with DS [29,30]. Thus, CMC–COP with a high DS appeared to accelerate the wound healing process.

#### 2.6.2. Wound Healing Rate

The wound area decreased over time in all groups, with an increasing healing rate. Table 2 shows the wound healing rate at different times after treatment. On day 3, as the wound area increased due to swelling, the healing rate was negative in the control and COP groups. On day 5, the wound area began to decrease, and the highest healing rate was observed in the CMC–COP and MEBO groups. On day 21, the healing rate was close to 100% in the CMC–COP and MEBO groups but was 89.42% and 90.88% in the control and COP groups, respectively. The healing rate of 99.93% in the CMC–COP group suggested complete epithelization and healing. The CMC–COP sponge was found to substantially improve wound healing, yielding a significantly higher healing rate than that of the control or COP groups on days 7 and 14 (** *p* < 0.01). The wound healing rate of CMC–COP was comparable to that of the MEBO group, indicating that the CMC–COP sponges were effective at promoting burn wound healing.

#### 2.6.3. Histological Evaluation

**Figure 8:**
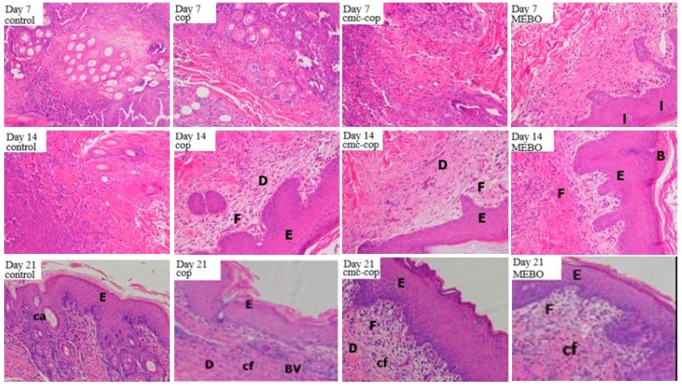
Micrographs of wound tissues in rabbits (H&E, 200×). Note: F, fibroblast; E, epidermis; D, dermis; Cf, collagen fiber; BV, blood vessel; Ca, cutaneous appendage; I, inflammatory; B, blister.

On day 1 post burn, the epidermis was severely damaged in all groups, accompanied by necrosis, edema, and massive inflammatory cell infiltration. Necrotic hair follicles were seen, the epidermis disappeared, and skin appendages contracted. On day 7, significant scarring and inflammatory cell infiltration were observed in all groups, and the skin at the edges of the wounds began to peel (Figure 8). On day 14, inflammatory cells were observed in the control and COP groups, whereas the wounds in the CMC–COP and MEBO groups were significantly re-epithelialized and the stratum corneum was reconstructed. On day 21, the dermal interstitial cells were compactly arranged, and the L929 cells were fusiform in the CMC–COP and MEBO groups, indicating sufficient recovery. In addition, inflammatory cells disappeared, and mature granulation tissue proliferation appeared in the dermis layer. The carboxymethyl chitosan spongy dressing had more ventilatory properties than MEBO, a burn cream commonly used in clinical practice, consistent with the report that carboxymethyl chitosan forms a polyelectric sponge at the wound, which binds to heparin or the platelet-derived growth factor in serum to protect them from enzymatic degradation, thereby indirectly stimulating fibroblast proliferation [31]. Overall, the histological findings showed that the CMC–COP sponge had beneficial effects on the pathological repair of tissue injury and wound healing.

## 3. Materials and Methods

### 3.1. Materials

The carboxymethyl chitosan (CMC, carboxylation >80%, analytical purity) was obtained from Shanghai Yuanye Biotechnology Co., Ltd. (Shanghai, China). The fish collagen peptide (COP) was prepared in our laboratory (*M*_w_920) [32]. The commercial burn cream (MEBO) was purchased from MEBO Pharmaceuticals Co., Ltd. (Shantou, China). N-hydroxysuccinimide (NHS) and 1-(3-dimethylaminopropyl)-3-ethylcarbodiimide hydrochloride (EDC) were purchased from the Sinopharm Chemical Reagent Company (Shanghai, China).

### 3.2. Preparation of the CMC–COP

Carboxymethyl chitosan (1.00 g) was dissolved in phosphate buffer solution (PBS, pH 6.0, 0.2 mol/L) and activated at a specified temperature for 2 h after the addition of 0.150 g of EDC and 0.150 g of NHS. Next, 1.00 g of collagen peptide dissolved in a buffer was added to the above reaction mixture. After magnetic stirring at the same temperature for a period of time, the reaction mixture was dialyzed for 3 d and freeze dried to obtain the CMC–COP sponges (Freeze dryer: FD-1A50, Bilon, Shanghai, China).

### 3.3. Determination of the Degree of Substitution (DS) of the CMC–COP

The DS of the CMC–COP was determined by UV spectrophotometry [33] (UV Spectrophotometer: UV1800PC, Lab-Spectrum, Shanghai, China). The maximum absorption wavelength of the collagen peptide was 200 nm. Hence, fish collagen peptide solutions were prepared at concentrations of 0.01, 0.015, 0.02, 0.025, and 0.03 g/L and measured for absorbance at 200 nm. A standard curve (Equation (1)) was obtained with a collagen peptide concentration as the abscissa. Based on the relationship between absorbance and concentration, the DS of the CMC–COP was calculated by Equation (2):

(1) $$A=44.08C+0.061,\;R^{2}=0.9998$$

(2) $$\mathrm{DS}=\frac{9800C}{920-46600C}\times 100\%$$

where A is the ultraviolet absorbance, C is the concentration of collagen g/L, and DS is the degree of substitution.

### 3.4. Appearance of CMC–COP

The CMC–COP sponges were photographed with a digital camera and the sponge appearance was observed by scanning electron microscopy (SEM, HitachiS-4800, Tokyo, Japan).

### 3.5. FTIR Analysis

The samples were ground into fine powder and then mixed with potassium bromide powder, ground, pressed into disks, and scanned over a wavelength range of 400–4000 cm^−1^ to obtain the infrared spectra (FTIR: Nicolet 170SX, Nicolet, Madison, WI, USA).

### 3.6. Cell Viability Assay

The cell viability assay was performed using the MTT method [34]. The L929 cells were seeded in 96-well culture plates (6000 cells/well) with 100 μL of DMEM containing 10% fetal bovine serum (FBS). After incubation for 24 h, the CMC–COP sponges were dissolved in PBS and added to the 96-well plates. After incubation for 22 h, 20 μL of the MTT solution (5 mg/mL) was added to each well, followed by incubation at 37 °C in darkness. After 4 h, 100 μL of dimethyl sulfoxide (DMSO) was added to each well, and the plates were then shaken for 10 min to dissolve the MTT formazan crystals. The optical density at 492 nm (OD492) was measured using a microplate reader. Cell viability was calculated according to the following equation:

(3) $$\mathrm{Cell}\;\mathrm{viability}\;\left(\%\right)={\mathrm{OD}492}_{\mathrm{sample}}/{\mathrm{OD}492}_{\mathrm{control}}\times 100\%$$

where OD492 (sample) and OD492 (control) are the OD values of the sample and control groups, respectively.

### 3.7. In Vitro Scratch Wound Healing Assay

Four groups, including a blank control, CMC–COP12, CMC–COP26, and CMC–COP58, were established. Fibroblasts (L929, 5 × l0^5^ cells/mL) in the logarithmic growth phase were seeded in 24-well culture plates. Scratches were made perpendicular to the horizontal line on the back of the wells when the cells reached >90% confluence. Subsequently, 1 mL of DMEM containing 10% FBS and each treatment was added to the corresponding wells, followed by incubation at 37 °C in a 5% CO_2_ incubator. Fields of view were randomly selected and photographed at 0, 12, 24, and 36 h (CKX41-A32PH, Olympus, Tokyo, Japan). Three parallel experiments were conducted.

### 3.8. Wound Healing Assay

#### 3.8.1. Animals

A total of 60 New Zealand rabbits, half female and half male, aged between 5 and 7 weeks, each weighing 1.5 to 1.8 kg, were provided by the Guangdong Medical Laboratory Animal Center (Foshan, China). The experiment was conducted in accordance with the guidelines and ethical standards set by the Laboratory Animal Care and Use Committee of Guangdong Medical University (SYXK20150147, 24 July 2015). The rabbits were given food and water and housed under a constant temperature of 25 ± 2 °C (12 h of the day–night cycle). Each group of 15 rabbits was treated with the control, COP, CMC–COP, and MEBO, respectively. The rabbits in the control group were treated with physiological saline only, and the drugs were changed every day.

#### 3.8.2. Burn Model

The rabbits were anesthetized, with their skins then de-haired and sterilized. Four scald wounds (3 cm^2^) were created from the lateral midline on the back using a scalding temperature control instrument (YLS-5Q). The burn device was used at a probe temperature of 100 °C, a working pressure of 1000 g, and a probe–skin contact time of 6 s. After burning, the rabbits were daily treated with 1 g of the physiological saline, COP, CMC–COP, or MEBO, respectively.

#### 3.8.3. Macroscopic Evaluation of Burn Wounds in Rabbits

Each group of animals was observed to monitor wound size, color, inflammation, drainage, infection, and the formation and separation of eschars on days 1, 7, 14, and 21 post burn. The wound area was measured by covering the back of rabbits with standard translucent weighing paper and cutting the paper into the shape of the wound. Wound healing was defined as no eschar or drainage on the surface of the wound. The wound healing rate was calculated according to the following equation:

Wound healing rate (%) = (S_0_ − S_1_)/S_0_ × 100% (4)

where S_0_ and S_1_ are the original and unhealed wound areas, respectively.

#### 3.8.4. Histological Evaluation of Burn Wounds in Rabbits

On days 1, 7, 14 and 21 after modeling, ten New Zealand rabbits were randomly selected (five males and five females) for sacrifice. The tissues around the wound were removed and fixed in a Bouin’s fixative for pathological sectioning. The sections were stained with hematoxylin and eosin (H&E) and observed under a fluorescence microscope (IX73, Olympus, Tokyo, Japan).

### 3.9. Statistical Analysis

Data were processed with an IBM SPSS Statistics v22 and analyzed using independent- samplet-tests. Numerical data are expressed as means ± SDs, and the differences among groups were analyzed using a one-way analysis of variance test. Here, *p* < 0.05 indicated statistical significance.

## 4. Conclusions

The CMC–COP sponges were synthesized in the aqueous phase using EDC and NHS as cross-linkers. In vitro tests showed that the CMC–COP sponges had excellent biological activities, including the promotion of cell viability and fibroblast migration. The scald wound healing experiments in rabbits demonstrated that CMC–COP sponges are an effective and promising agent for burn care.

## Figures and Tables

**Table 1:** Amino acid composition of collagen peptide.

NO.	Amino Acid	Content (g/100 g)
1	ASP	5.46
2	THR	2.84
3	SER	3.61
4	GLU	10.7
5	GLY	22.1
6	ALA	9.77
7	VAL	2.47
8	MET	1.75
9	ILEl	1.35
10	LEU	2.68
11	TYR	0.71
12	PHE	1.68
13	HIS	0.94
14	LYS	3.31
15	ARG	6.94
16	PRO	11.3
17	Hyp	11.85
	Free amino acid	0.54
Total		100

**Table 2:** Wound healing rates of the COP, CMC–COP, and MEBO groups (%, *n* = 10).

Group	Day 3	Day 5	Day 7	Day 14	Day 21
Control	−1.53 ± 0.55	3.56 ± 1.80	8.90 ± 1.03	35.47 ± 1.77	89.42 ± 1.96
COP	−0.73 ± 0.61	7.86 ± 1.88	13.17 ± 0.92 **	46.29 ± 0.84 **	90.88 ± 0.22
CMC–COP	1.71 ± 0.53	9.82 ± 1.72	19.59 ± 0.78 **	66.97 ± 1.31 **	99.93 ± 0.15
MEBO	1.76 ± 0.42	9.10 ± 1.18	24.16 ± 1.18 **	72.65 ± 0.57 **	99.97 ± 0.07

** *p* < 0.01 compared with control group.
